# Synthesis and crystal structure of the mixed-valent Te^IV/VI^ compound PbTe_3_O_8_

**DOI:** 10.1107/S2056989026010996

**Published:** 2026-10-30

**Authors:** Matthias Weil

**Affiliations:** a Institute for Chemical Technologies and Analytics, Division of Applied Solid State Chemistry, TU Wien, Getreidemarkt 9/E164-05-1, A-1060, Vienna, Austria; National Taras Shevchenko University of Kyiv, Ukraine

**Keywords:** crystal structure, isotypism, mixed-valent compound, structural comparison

## Abstract

The crystal structure of the mixed-valent compound PbTe_3_O_8_(= Pb^II^Te^IV^_2_Te^VI^O_8_), lead(II) bis­[oxidotellurate(IV)] tellurate(VI), is isotypic with that of SrTe_3_O_8_. It comprises ^1^_∞_[Te_3_O_8_]^2–^ chains sharing O atoms to form a framework with two types of square channels extending parallel to [001]. The smaller channels (diameter ∼4 Å) are occupied by 12-coordinate Pb^II^ atoms in the centre of the channel, whereas the slightly larger channels (diameter ∼4.5 Å) accommodate the stereoactive 5*s*^2^ electron lone pair of Te^IV^.

## Abstract 2

PbTe_3_O_8_ is a mixed-valent Te^IV/VI^ compound isotypic with SrTe_3_O_8_.

## 1. Chemical context

In addition to the inter­est in the crystal-chemical peculiarities of compounds containing Pb^II^ and Te^IV^ atoms that are characterized by their lone pair electrons (Walsh *et al.*, 2011 ▸), phases in the Pb/Te/O system are also relevant in terms of application-related problems. This applies, for example, to PbTe-based thermoelectrics (Kanatzidis, 2010 ▸) or to Pb/Te/O-based high-refractive index glasses (Alazoumi *et al.*, 2018 ▸), where they can occur as unwanted surfacial oxidation products and as (re)crystallized phases, respectively. It is therefore important to have a thorough knowledge of the formation conditions and crystal structures of such phases.

Taking lead(II) oxidotellurate(VI), PbTeO_4_, as an example, polycrystalline material from it was prepared at a temperature of 973 K and a pressure of 3000 atm (Sleight *et al.*, 1972 ▸). In an attempt to grow PbTeO_4_ crystals under more moderate hydro­thermal conditions following a modified procedure reported by Mathers & Graham (1929 ▸), crystals of the so far unknown compound PbTe_3_O_8_ were obtained serendipitously, and its crystal structure is reported in the present communication. As it turned out, PbTeO_4_ can only be prepared at higher pressure and temperature conditions (Hladik *et al.*, 2024 ▸).

## 2. Structural commentary

PbTe_3_O_8_ expands our knowledge of ternary phases in the Pb/Te/O system (all contain lead solely in oxidation state +II), of which the following are currently known. For oxidotellurates(IV): *α*-PbTeO_3_ (Mariolacos, 1969 ▸; Zavodnik *et al.*, 2008 ▸; Missen *et al.*, 2019*b* ▸), *β*-PbTeO_3_ (Sciau *et al.*, 1986 ▸; Ma *et al.*, 2017 ▸; Weil *et al.*, 2018 ▸), *γ*-PbTeO_3_ (Weil *et al.*, 2018 ▸; Kampf *et al.*, 2022 ▸), Pb_2_Te_3_O_8_ (Dewan *et al.*, 1978 ▸; Champarnaud-Mesjard *et al.*, 2001 ▸), PbTe_5_O_11_ (Oufkir *et al.*, 2001 ▸); for oxidotellurates(VI): PbTeO_4_ (Hladik *et al.*, 2024 ▸), Pb_2_TeO_5_ (Kampf *et al.*, 2010 ▸; Artner & Weil, 2013 ▸), Pb_5_TeO_8_ (Artner & Weil, 2013 ▸). This makes PbTe_3_O_8_ the first mixed-valent Te^IV/VI^ compound (= Pb^II^Te^IV^_2_Te^VI^O_8_) in the ternary Pb/Te/O system. It belongs to class I of the Robin & Day (1967 ▸) classification, with the different valence states each clearly located on their own atomic sites.

The crystal structure of PbTe_3_O_8_ is isotypic with that of SrTe_3_O_8_ (Barrier *et al.*, 2006 ▸; Weil & Stöger, 2007 ▸). It can be considered as being composed of _∞_^1^[Te_2_^IV^Te^VI^O_8_]^2–^ chains, with adjacent chains sharing O atoms to form a framework with two types of square channels extending parallel to [001] (Fig. 1 ▸).

The _∞_^1^[Te_2_^IV^Te^VI^O_8_]^2–^ chains consist of {Te1_2_O_8_} double polyhedra, which are formed by two symmetry-related {Te1^IV^O_5_} pyramids (Te1, site symmetry *m*..), and of {Te2^VI^O_6_} octa­hedra (Te2, 2/*m*..) by sharing corners and edges (Fig. 2 ▸). In the framework, each {Te2O_6_} octa­hedron is connected to four {Te1_2_O_8_} double polyhedra, two *via* shared edges and two *via* shared corners, and each {Te1_2_O_8_} double polyhedron is connected in the same way to four {Te2O_6_} octa­hedra. The {Te1O_5_} pyramid deviates somewhat from an ideal square-pyramidal coordination geometry (the τ_5_ index is 0.23, with extreme values of 0 for ideal square-pyramidal and 1 for ideal trigonal-bipyramidal geometry; Addison *et al.*, 1984 ▸). The Te—O bond lengths span a range from 1.948 (10) to 2.170 (9) Å (Table 1 ▸). The {Te2O_6_} octa­hedron shows no variance in bond lengths (Table 1 ▸) and only minor angular distortions for the equatorial O atoms [range 85.2 (4)–94.8 (4)°]. All Te—O bond lengths are in characteristic ranges for tetra- and hexa­valent tellurium atoms (Christy *et al.*, 2016 ▸). The *LPLoc* software (Hamani *et al.*, 2020 ▸) revealed the position of the presumed 5*s*^2^ electron lone pair at the Te1^IV^ site with resulting fractional coordinates of *x* = 0.49158, *y* = 0.35089, *z* = 0. The radius of the electron lone pair was calculated to be 1.03 Å with a distance of 1.027 Å from the Te1 position. The electron lone pair protrudes into the larger of the two channels (diameter ∼4.5 Å), and thus is stereochemically active, as shown in Fig. 1 ▸.

The Pb^II^ cation is located in the smaller channel (diameter ∼4 Å, Fig. 1 ▸). It exhibits site symmetry 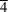.. and has a coordination number of 12, with three groups of short, medium and long Pb—O bond lengths (Table 1 ▸). The corresponding [PbO_12_] coordination polyhedron was analysed using the *Polynator* program (Link & Niewa, 2023 ▸). It has a volume of 52.82 Å^3^ and can be described as a distorted cubocta­hedron that differs from the ideal polyhedron (point group 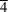
2*m*) by a δ value (Link & Niewa, 2023 ▸) of 5.736. The average Pb^[12]^—O distance (2.853 Å) is in good agreement with the value of 2.827 (113) Å provided by Gagné & Hawthorne (2018 ▸). Localization of the electron lone pair at the Pb^II^ site using *LPLoc* shows that it is situated directly at the metal site (calculated lone pair radius 1.16 Å) and apparently exhibits no stereochemical activity.

A qu­anti­tative comparison between the isotypic *M*Te_3_O_8_ (*M* = Pb, Sr) structures was carried out using the *compstru* software (de la Flor *et al.*, 2016 ▸), available at the Bilbao Crystallographic Server (Aroyo *et al.*, 2006 ▸). The absolute distances between paired atoms are 0 Å for Pb1/Sr1, 0 Å for Te2, 0.0235 Å for Te1, 0.0247 Å for O3, 0.0252 Å for O1, and 0.0600 Å for O2; the arithmetic mean of the distance between paired atoms is 0.0264 Å. The degree of lattice distortion (0.0070), and the low value for the measure of similarity (0.024) indicate a high degree of simiularity for the two crystal structures.

In addition to the two isotypic *M*Te_3_O_8_ (*M* = Pb, Sr) structures, there is another compound with the same formula type (*M* = Ca) that occurs in nature as the rare mineral carlfriesite. Its monoclinic crystal structure (Effenberger *et al.*, 1978 ▸; Missen *et al.*, 2019*a* ▸) can also be described as a nanoporous framework, although it exhibits a different topology for the [Te_2_^IV^Te^VI^O_8_]^2–^ anion. Here, the metal cation is situated in slightly larger channels with a diameter of approximately 6 Å and exhibits a coordination number of eight with a distorted tetra­gonal anti­prismatic oxygen coordination.

## 3. Synthesis and crystallization

The chemicals were supplied by Merck and were used without further purification. Equimolar amounts of PbO_2_ and TeO_2_ (total load 0.3 g) were thoroughly mixed and placed in a Teflon container. The container was filled to about two-thirds of its inner volume (5 ml) with water, closed with a Teflon lid and placed in a steel autoclave, which was heated at 483 K for two days. The greyish solid product was filtered off and washed with water and ethanol. A few colourless crystals with a rod-like form of the title compound were isolated under a polarising microscope. Qualitative phase analysis of the bulk product from laboratory X-ray powder diffraction data revealed the starting materials TeO_2_ and PbO_2_ as well as PbTe_3_O_8_ as products, together with unassigned reflections. This shows that, in a redox reaction, parts of Pb^IV^ and Te^IV^ have reacted to form Pb^II^ and Te^VI^.

## 4. Refinement

Crystal data, data collection and structure refinement details are summarized in Table 2 ▸. For better comparison with isotypic SrTe_3_O_8_ (Weil & Stöger, 2007 ▸), the same atom numbering scheme and starting coordinates for refinement were used. The residual maximum and minimum difference-electron densities are located 0.61 Å from Te2 and 0.49 Å from Pb1, respectively.

Compared with the other atoms, the displacement parameters of the Pb site are large. The same phenomenon is observed in the isotypic Sr structure (Weil & Stöger, 2007 ▸). We assume that this property is characteristic of this type of structure and is primarily caused by the position of the metal site within the large pores. It is also possible that the Pb site shows positional order around the 4 axis or is under-occupied with a potential Pb^II/IV^ co-occupation, but this cannot be conclusively demonstrated by the available X-ray data. To obtain a more detailed picture, supplementary analytical data are required, for example through wet chemical methods or microprobe analysis.

## Supplementary Material

Crystal structure: contains datablock(s) I, global. DOI: 10.1107/S2056989026010996/nu2020sup1.cif

Structure factors: contains datablock(s) I. DOI: 10.1107/S2056989026010996/nu2020Isup2.hkl

CCDC reference: 2597291

Additional supporting information:  crystallographic information; 3D view; checkCIF report

## Figures and Tables

**Figure 1:**
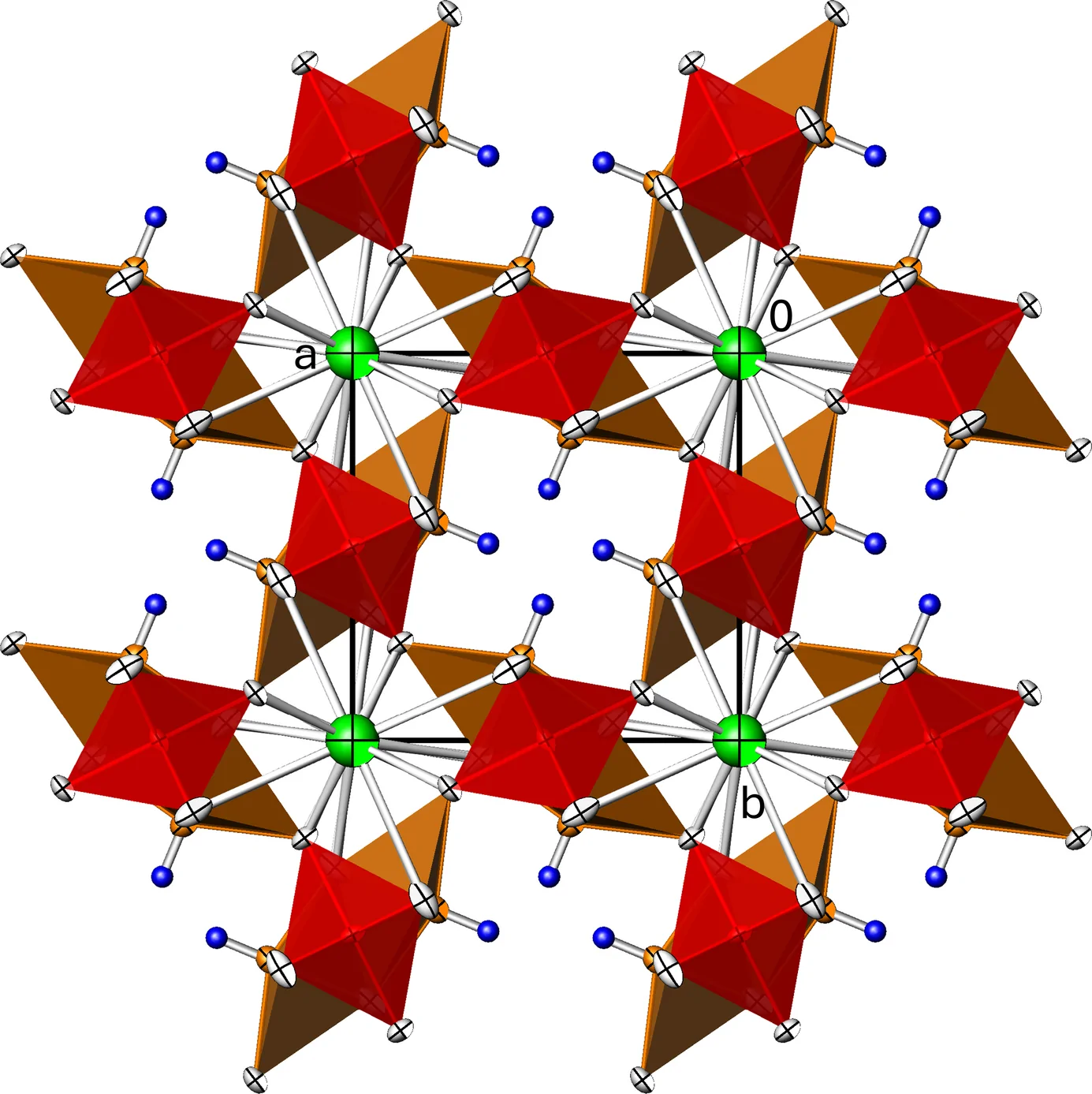
View of the crystal structure of PbTe_3_O_8_ in a projection along [001]. Displacement ellipsoids are drawn at the 90% probability level. Colour code: Pb green, O colourless, {Te1O_5_} square pyramid orange, {Te2O_6_} octa­hedron red; the 5*s*^2^ lone pair at the Te1 site is indicated as a blue sphere of arbitrary size.

**Figure 2:**
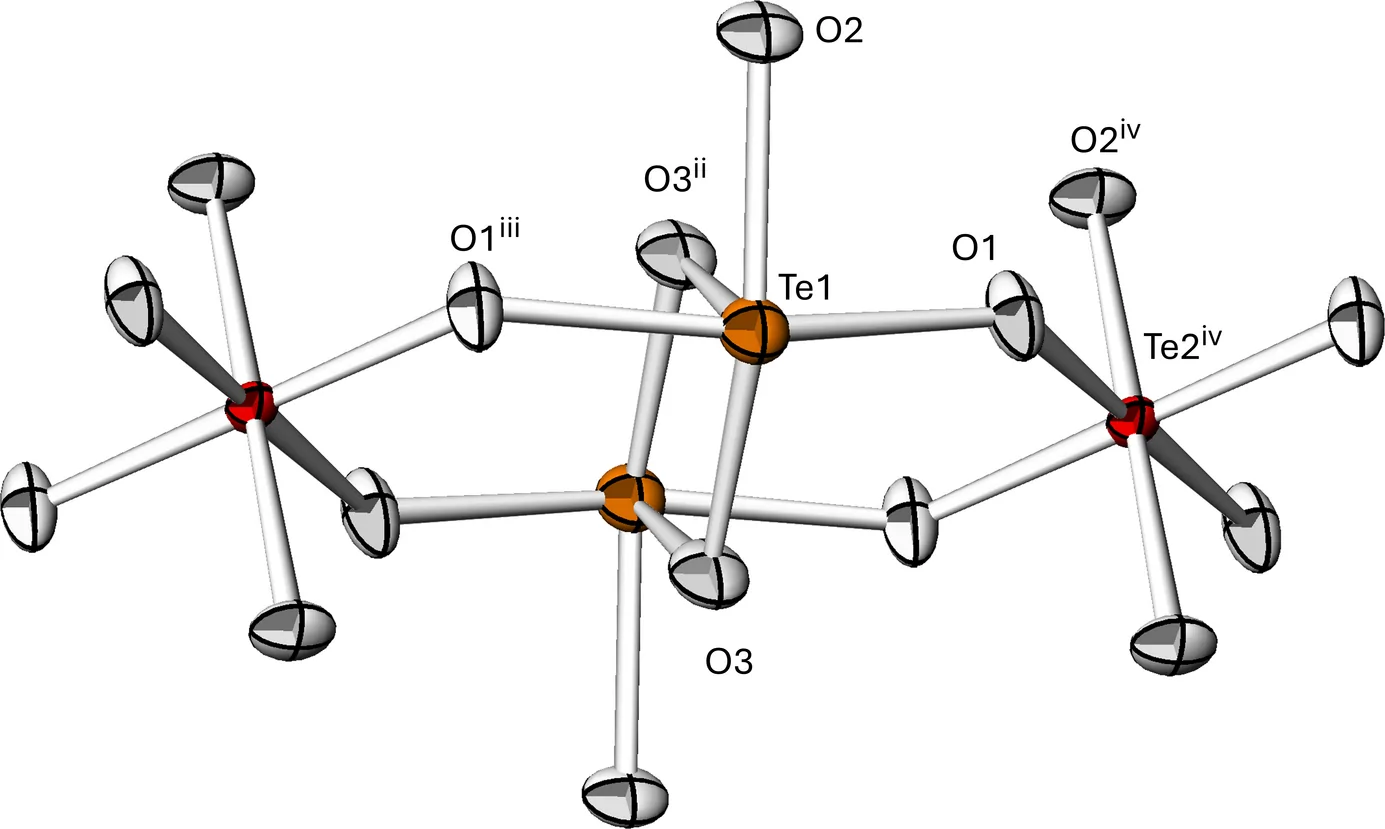
Part of a _∞_^1^[Te_2_^IV^Te^VI^O_8_]^2–^ chain. Displacement ellipsoids are drawn at the 90% probability level. Symmetry codes refer to Table 1 ▸.

**Table 1:** Selected bond lengths (Å)

Pb1—O2	2.570 (7)	Te1—O1	2.080 (7)
Pb1—O3^i^	2.842 (8)	Te1—O1^iii^	2.080 (7)
Pb1—O1	3.147 (8)	Te1—O2	2.170 (9)
Te1—O3^ii^	1.948 (10)	Te2—O1^iv^	1.927 (7)
Te1—O3	2.027 (9)	Te2—O2	1.927 (9)

Symmetry codes: (i) 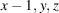; (ii) 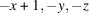; (iii) 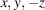; (iv) 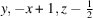.

**Table 2:** Experimental details

Crystal data	
Chemical formula	PbTe_3_O_8_
*M*_r_	717.99
Crystal system, space group	Tetragonal, *P*4_2_/*m*
Temperature (K)	296
*a*, *c* (Å)	6.9345 (3), 6.7369 (4)
*V* (Å^3^)	323.96 (3)
*Z*	2
Radiation type	Mo *K*α
μ (mm^−1^)	39.29
Crystal size (mm)	0.10 × 0.02 × 0.01

Data collection	
Diffractometer	Bruker APEXII CCD
Absorption correction	Multi-scan (*SADABS*; Krause *et al.*, 2015 ▸)
*T*_min_, *T*_max_	0.155, 0.349
No. of measured, independent and observed [*I* > 2σ(*I*)] reflections	7237, 514, 499
*R*_int_	0.031
(sin θ/λ)_max_ (Å^−1^)	0.702

Refinement	
*R*[*F*^2^ > 2σ(*F*^2^)], *wR*(*F*^2^), *S*	0.037, 0.112, 1.12
No. of reflections	514
No. of parameters	34
Δρ_max_, Δρ_min_ (e Å^−3^)	3.82, −4.85

Computer programs: *APEX3* and *SAINT* (Bruker, 2018 ▸), *SHELXL* (Sheldrick, 2015 ▸), *ATOMS* (Dowty, 2006 ▸) and *publCIF* (Westrip, 2010 ▸).

## supplementary crystallographic information

### Lead(II) bis[oxidotellurate(IV)] tellurate(VI) Crystal data

**Table:**

PbTe_3_O_8_	*D*_x_ = 7.360 Mg m^−^^3^
*M**_r_* = 717.99	Mo *K*α radiation, λ = 0.71073 Å
Tetragonal, *P*4_2_/*m*	Cell parameters from 4370 reflections
*a* = 6.9345 (3) Å	θ = 3.0–29.4°
*c* = 6.7369 (4) Å	µ = 39.29 mm^−^^1^
*V* = 323.96 (3) Å^3^	*T* = 296 K
*Z* = 2	Fragment, colourless
*F*(000) = 604	0.10 × 0.02 × 0.01 mm

### Lead(II) bis[oxidotellurate(IV)] tellurate(VI) Data collection

**Table:**

Bruker APEXII CCD diffractometer	499 reflections with *I* > 2σ(*I*)
ω– and φ–scans	*R*_int_ = 0.031
Absorption correction: multi-scan (*SADABS*; Krause *et al.*, 2015)	θ_max_ = 29.9°, θ_min_ = 2.9°
*T*_min_ = 0.155, *T*_max_ = 0.349	*h* = −9→9
7237 measured reflections	*k* = −9→9
514 independent reflections	*l* = −9→9

### Lead(II) bis[oxidotellurate(IV)] tellurate(VI) Refinement

**Table:**

Refinement on *F*^2^	0 restraints
Least-squares matrix: full	Primary atom site location: isomorphous structure methods
*R*[*F*^2^ > 2σ(*F*^2^)] = 0.037	*w* = 1/[σ^2^(*F*_o_^2^) + (0.0619*P*)^2^ + 13.4507*P*] where *P* = (*F*_o_^2^ + 2*F*_c_^2^)/3
*wR*(*F*^2^) = 0.112	(Δ/σ)_max_ < 0.001
*S* = 1.12	Δρ_max_ = 3.82 e Å^−^^3^
514 reflections	Δρ_min_ = −4.85 e Å^−^^3^
34 parameters	

### Lead(II) bis[oxidotellurate(IV)] tellurate(VI) Special details

**Table:**

Geometry. All esds (except the esd in the dihedral angle between two l.s. planes) are estimated using the full covariance matrix. The cell esds are taken into account individually in the estimation of esds in distances, angles and torsion angles; correlations between esds in cell parameters are only used when they are defined by crystal symmetry. An approximate (isotropic) treatment of cell esds is used for estimating esds involving l.s. planes.

### Lead(II) bis[oxidotellurate(IV)] tellurate(VI) Fractional atomic coordinates and isotropic or equivalent isotropic displacement parameters (Å^2^)

**Table:**

	*x*	*y*	*z*	*U*_iso_*/*U*_eq_	
Pb1	0.000000	0.000000	0.250000	0.0409 (4)	
Te1	0.43446 (12)	0.21418 (12)	0.000000	0.0105 (3)	
Te2	0.000000	0.500000	0.000000	0.0066 (3)	
O1	0.4112 (10)	0.1842 (10)	0.3063 (10)	0.0125 (12)	
O2	0.1237 (14)	0.2511 (13)	0.000000	0.0111 (17)	
O3	0.6731 (15)	0.0454 (14)	0.000000	0.0129 (18)	

### Lead(II) bis[oxidotellurate(IV)] tellurate(VI) Atomic displacement parameters (Å^2^)

**Table:**

	*U*^11^	*U*^22^	*U*^33^	*U*^12^	*U*^13^	*U*^23^
Pb1	0.0373 (4)	0.0373 (4)	0.0480 (7)	0.000	0.000	0.000
Te1	0.0105 (4)	0.0101 (4)	0.0109 (4)	0.0020 (3)	0.000	0.000
Te2	0.0086 (5)	0.0052 (5)	0.0061 (5)	0.0025 (3)	0.000	0.000
O1	0.019 (3)	0.012 (3)	0.007 (3)	0.007 (2)	0.001 (2)	0.001 (2)
O2	0.009 (4)	0.007 (4)	0.017 (4)	0.003 (3)	0.000	0.000
O3	0.012 (4)	0.013 (4)	0.014 (4)	0.007 (3)	0.000	0.000

### Lead(II) bis[oxidotellurate(IV)] tellurate(VI) Geometric parameters (Å, º)

**Table:**

Pb1—O2^i^	2.570 (7)	Te1—O3^vii^	1.948 (10)
Pb1—O2^ii^	2.570 (7)	Te1—O3	2.027 (9)
Pb1—O2^iii^	2.570 (7)	Te1—O1	2.080 (7)
Pb1—O2	2.570 (7)	Te1—O1^x^	2.080 (7)
Pb1—O3^iv^	2.842 (8)	Te1—O2	2.170 (9)
Pb1—O3^v^	2.842 (8)	Te2—O1^iv^	1.927 (7)
Pb1—O3^vi^	2.842 (8)	Te2—O1^xi^	1.927 (7)
Pb1—O3^vii^	2.842 (8)	Te2—O1^ix^	1.927 (7)
Pb1—O1	3.147 (8)	Te2—O1^xii^	1.927 (7)
Pb1—O1^viii^	3.147 (8)	Te2—O2	1.927 (9)
Pb1—O1^ii^	3.147 (7)	Te2—O2^xiii^	1.927 (9)
Pb1—O1^ix^	3.147 (7)		
			
O2^i^—Pb1—O2^ii^	115.43 (15)	O1—Pb1—O1^ii^	90.83 (3)
O2^i^—Pb1—O2^iii^	115.43 (15)	O1^viii^—Pb1—O1^ii^	90.83 (3)
O2^ii^—Pb1—O2^iii^	98.1 (3)	O2^i^—Pb1—O1^ix^	113.5 (2)
O2^i^—Pb1—O2	98.1 (3)	O2^ii^—Pb1—O1^ix^	131.0 (2)
O2^ii^—Pb1—O2	115.43 (15)	O2^iii^—Pb1—O1^ix^	60.1 (2)
O2^iii^—Pb1—O2	115.43 (15)	O2—Pb1—O1^ix^	56.1 (2)
O2^i^—Pb1—O3^iv^	165.0 (3)	O3^iv^—Pb1—O1^ix^	52.7 (2)
O2^ii^—Pb1—O3^iv^	78.6 (3)	O3^v^—Pb1—O1^ix^	60.3 (2)
O2^iii^—Pb1—O3^iv^	54.6 (3)	O3^vi^—Pb1—O1^ix^	138.5 (2)
O2—Pb1—O3^iv^	79.10 (19)	O3^vii^—Pb1—O1^ix^	110.6 (2)
O2^i^—Pb1—O3^v^	54.6 (3)	O1—Pb1—O1^ix^	90.83 (3)
O2^ii^—Pb1—O3^v^	165.0 (3)	O1^viii^—Pb1—O1^ix^	90.83 (3)
O2^iii^—Pb1—O3^v^	79.10 (19)	O1^ii^—Pb1—O1^ix^	166.2 (3)
O2—Pb1—O3^v^	78.6 (3)	O3^vii^—Te1—O3	77.2 (5)
O3^iv^—Pb1—O3^v^	110.57 (12)	O3^vii^—Te1—O1	83.0 (2)
O2^i^—Pb1—O3^vi^	79.10 (19)	O3—Te1—O1	90.32 (18)
O2^ii^—Pb1—O3^vi^	54.6 (3)	O3^vii^—Te1—O1^x^	83.0 (2)
O2^iii^—Pb1—O3^vi^	78.6 (3)	O3—Te1—O1^x^	90.32 (18)
O2—Pb1—O3^vi^	165.0 (3)	O1—Te1—O1^x^	165.5 (4)
O3^iv^—Pb1—O3^vi^	107.3 (2)	O3^vii^—Te1—O2	74.3 (4)
O3^v^—Pb1—O3^vi^	110.57 (12)	O3—Te1—O2	151.5 (4)
O2^i^—Pb1—O3^vii^	78.6 (3)	O1—Te1—O2	86.3 (2)
O2^ii^—Pb1—O3^vii^	79.10 (19)	O1^x^—Te1—O2	86.3 (2)
O2^iii^—Pb1—O3^vii^	165.0 (3)	O1^iv^—Te2—O1^xi^	180.0
O2—Pb1—O3^vii^	54.6 (3)	O1^iv^—Te2—O1^ix^	94.8 (4)
O3^iv^—Pb1—O3^vii^	110.57 (12)	O1^xi^—Te2—O1^ix^	85.2 (4)
O3^v^—Pb1—O3^vii^	107.3 (2)	O1^iv^—Te2—O1^xii^	85.2 (4)
O3^vi^—Pb1—O3^vii^	110.57 (12)	O1^xi^—Te2—O1^xii^	94.8 (4)
O2^i^—Pb1—O1	131.0 (2)	O1^ix^—Te2—O1^xii^	180.0 (4)
O2^ii^—Pb1—O1	56.1 (2)	O1^iv^—Te2—O2	89.5 (3)
O2^iii^—Pb1—O1	113.5 (2)	O1^xi^—Te2—O2	90.5 (3)
O2—Pb1—O1	60.1 (2)	O1^ix^—Te2—O2	90.5 (3)
O3^iv^—Pb1—O1	60.3 (2)	O1^xii^—Te2—O2	89.5 (3)
O3^v^—Pb1—O1	138.5 (2)	O1^iv^—Te2—O2^xiii^	90.5 (3)
O3^vi^—Pb1—O1	110.6 (2)	O1^xi^—Te2—O2^xiii^	89.5 (3)
O3^vii^—Pb1—O1	52.7 (2)	O1^ix^—Te2—O2^xiii^	89.5 (3)
O2^i^—Pb1—O1^viii^	60.1 (2)	O1^xii^—Te2—O2^xiii^	90.5 (3)
O2^ii^—Pb1—O1^viii^	113.5 (2)	O2—Te2—O2^xiii^	180.0
O2^iii^—Pb1—O1^viii^	56.1 (2)	Te2^xiv^—O1—Te1	135.5 (3)
O2—Pb1—O1^viii^	131.0 (2)	Te2^xiv^—O1—Pb1	95.9 (3)
O3^iv^—Pb1—O1^viii^	110.6 (2)	Te1—O1—Pb1	89.5 (2)
O3^v^—Pb1—O1^viii^	52.7 (2)	Te2—O2—Te1	123.2 (5)
O3^vi^—Pb1—O1^viii^	60.3 (2)	Te2—O2—Pb1^i^	117.3 (3)
O3^vii^—Pb1—O1^viii^	138.5 (2)	Te1—O2—Pb1^i^	104.6 (3)
O1—Pb1—O1^viii^	166.2 (3)	Te2—O2—Pb1	117.3 (3)
O2^i^—Pb1—O1^ii^	56.1 (2)	Te1—O2—Pb1	104.6 (3)
O2^ii^—Pb1—O1^ii^	60.1 (2)	Pb1^i^—O2—Pb1	81.9 (3)
O2^iii^—Pb1—O1^ii^	131.0 (2)	Te1^vii^—O3—Te1	102.8 (5)
O2—Pb1—O1^ii^	113.5 (2)	Te1^vii^—O3—Pb1^xv^	101.7 (3)
O3^iv^—Pb1—O1^ii^	138.5 (2)	Te1—O3—Pb1^xv^	135.7 (3)
O3^v^—Pb1—O1^ii^	110.6 (2)	Te1^vii^—O3—Pb1^vii^	101.7 (3)
O3^vi^—Pb1—O1^ii^	52.7 (2)	Te1—O3—Pb1^vii^	135.7 (3)
O3^vii^—Pb1—O1^ii^	60.3 (2)	Pb1^xv^—O3—Pb1^vii^	72.7 (2)

Symmetry codes: (i) −*x*, −*y*, −*z*; (ii) *y*, −*x*, −*z*+1/2; (iii) −*y*, *x*, *z*+1/2; (iv) *y*, −*x*+1, −*z*+1/2; (v) *x*−1, *y*, *z*; (vi) −*y*, *x*−1, *z*+1/2; (vii) −*x*+1, −*y*, −*z*; (viii) −*x*, −*y*, *z*; (ix) −*y*, *x*, −*z*+1/2; (x) *x*, *y*, −*z*; (xi) −*y*, *x*, *z*−1/2; (xii) *y*, −*x*+1, *z*−1/2; (xiii) −*x*, −*y*+1, −*z*; (xiv) −*y*+1, *x*, *z*+1/2; (xv) *x*+1, *y*, *z*.
